# Novel Lung Targeting Cell Penetrating Peptides as Vectors for Delivery of Therapeutics

**DOI:** 10.21203/rs.3.rs-1056707/v1

**Published:** 2021-11-15

**Authors:** Kayla McCandless, Sanjay Mishra, Jeffrey Stiltner, Kyle S. Feldman, Hisato Yagi, Raymond Yurko, Kazi Islam, Jonathan M. Brown, Raymond Frizzell, Maliha Zahid

**Affiliations:** 1 Dept. of Developmental Biology, University of Pittsburgh School of Medicine, Pittsburgh, PA; 2 Dept. of Pediatrics, University of Pittsburgh, School of Medicine, Pittsburgh, PA; 3 Peptide and Peptoid Synthesis Facility, University of Pittsburgh, PA; 4 Consultant, to MPEG LA, L.L.C, MD; 5 Clinical and Translational Science Institute, University of Pittsburgh, PA

**Keywords:** cell penetrating peptides, lung targeting peptides, S7A, R11A

## Abstract

Cell penetrating peptides are unique, 5–30 amino acid long peptides that are able to breach cell membrane barriers and carry cargoes intracellularly in a functional form. Our prior work identified a synthetic, non-naturally occurring 12-amino acid long peptide that we termed cardiac targeting peptide (CTP: APWHLSSQYSRT) due to its ability to transduce cardiomyocytes *in vivo*. Studies looking into its mechanism of transduction identified two lung targeting peptides (LTPs), S7A (APWHLSAQYSRT) and R11A (APWHLSSQYSAT). These peptides robustly transduced human bronchial epithelial cell lines *in vitro* and mouse lung tissue *in vivo*. This uptake occurred independently of clathrin mediated endocytosis. Biodistribution studies of R11A showed peak uptake at 15 minutes with uptake in liver but not kidneys, indicating primarily a hepatobiliary mode of excretion. Cyclic version of both peptides was ~100-fold more efficient in permeating cells than their linear counterparts. As proof of principle, we conjugated anti-spike and anti-envelope SARS-CoV-2 siRNAs to cyclized R11A and demonstrate anti-viral efficacy *in vitro*. Our work presented here identifies two novel lung-specific cell penetrating peptides that could potentially deliver myriad therapeutic cargoes to lung tissue.

## Introduction

Cell penetrating peptides (CPPs), also known as protein transduction domains, are 5–30 amino acid long peptides, capable of breaching cell membrane barriers while carrying cargoes much larger than themselves in an intact, functional form^1,2^. The first CPP, identified more than thirty years ago, was from the HIV coat protein, trans-activator of transcription (HIV-1 Tat), shown, in two simultaneously published papers, as having the ability to cross cell membrane barriers without a need for transfection reagents^3,4^. On the footsteps of these findings came the report of the third helix of Antennapedia domain as having transduction capabilities^5^. These abilities were mapped out to short 11–13 amino acid, cationic, arginine- and lysine-rich domains of the proteins. Not only were these peptides able to internalize full-length proteins and virus particles (e.g. HIV viral particle in the case of Tat), but they could be fused to the marker protein beta-galactosidase, and internalize not only into cells, but also cross the blood-brain barrier to transduce neuronal tissue^6^. However, this widespread, robust and generalized transduction of all tissue types after an intra-peritoneal injection^6^, was an obstacle to the development of these non-tissue specific CPPs as vectors for clinical use. This drawback was circumvented by the use of phage display, a Nobel prize in chemistry winning technique, developed by Smith^7^. Using this technique^8^, investigators have identified peptides targeting tumors^9^, pancreatic islet cells^10,11^, adipose tissue^12^, synovial tissue^13^ and cardiomyocytes^14^ to name a few examples.

Our prior work utilizing an *in vitro* and *in vivo* phage display methodology^8^ led to the identification of a unique, non-naturally occurring, 12-amino acid long cardiac targeting peptide (CTP)^14^ that was able to robustly transduce cardiomyocytes in as little as 15minutes after a peripheral, intra-venous injection^15^. This transduction ability was cardiomyocyte-specific and not species-limited, as demonstrated by us^15^, and others^16,17^. Studies into the mechanism of transduction of this peptide serendipitously identified two robust, lung targeting peptides (LTPs). In the current publication, we will detail the work leading to identification of these LTPs, biodistribution after intravenous administration, compare linear versus cyclized versions, and provide an application as vectors for delivery of siRNA targeting SARS-CoV-2 spike S and envelope E proteins.

## Results

**Figure 1:**
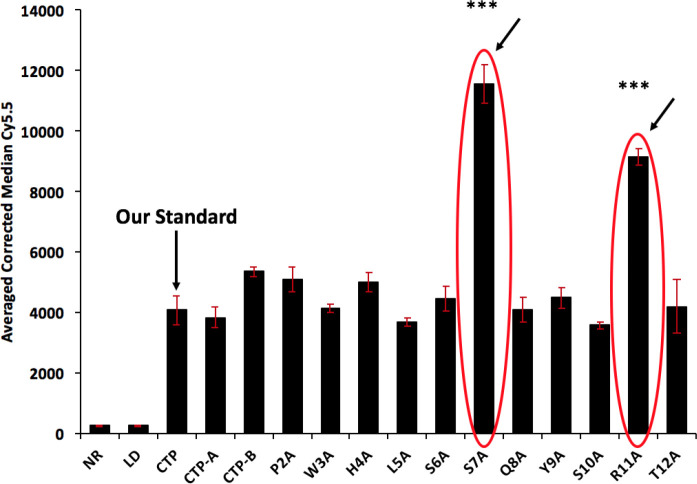
FACs of H9C2 cells incubated with fluorescently labeled (Cy5.5) alanine variants of CTP. Cy5.5 intensities are almost 3-fold higher in the S7A and R11A versions. All cell work done in biological triplicates. Error bars represent standard deviations. ***p<0.001

As part of a larger set of studies investigating the mechanism of transduction of CTP (APWHLSSQYSRT), we performed an alanine scan. Briefly peptides were synthesized using solid-phase peptide synthesis with each amino acid replaced sequentially with an alanine resulting in 11 variant peptides. The original 12-amino acid CTP was synthesized as a reference along with the 6 N-terminus CTP-A (APWHLS) and 6 C-terminus CTP-B (SQYSRT). All peptides were fluorescently labeled at the N-terminus with Cyanine 5.5 (Cy5.5), and amide-capped at the C-terminus. Rat cardiomyoblast H9C2 cells were incubated with 10μM of the peptides for 30 minutes at 37°C, washed extensively with PBS, trypsinized, fixed with formalin and fluorescence activated cell sorting (FACs) performed (Figure 1).

**Figure 2:**
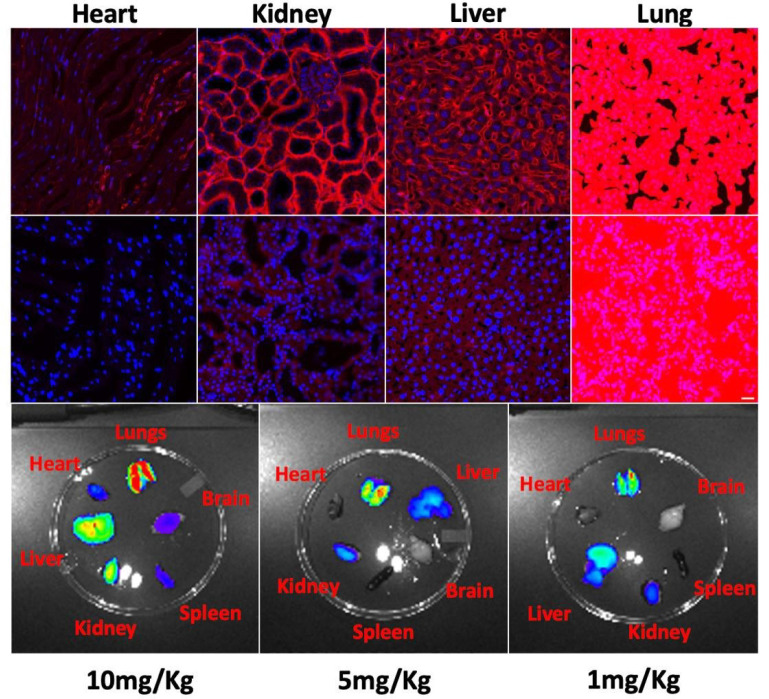
Uptake of fluorescently (Cy5.5) labeled S7A (top row) and R11A (middle row), 10mg/Kg, by heart, kidney, liver, and lung tissue in wild-type mice injected intravenously at 15 mins. First three panels imaged using same parameters-lungs imaged with significantly shorter exposure times to avoid saturation. Bottom Panel-Mice injected with decreasing doses of R11A, euthanized at 15 mins and ex vivo imaging of multiple organs performed using IVIS imaging system. There was robust lung uptake of R11A even at the lowest 1mg/Kg dose. N=3. Red-Peptides; Blue-Dapi. Scale bar represents 20μm.

The two peptides that most robustly transduced H9C2 cells, S7A (APWHLSAQYSRT) and R11A (APWHLSSQYSAT), were administered intravenously to wild-type CD1 mice (Charles Rivers Labs) at a dose of 10mg/Kg. After 30 minutes of circulation time, the mice were euthanized using inhalational CO2, chests opened, and organs perfusion fixed by placing a small nick in the right atrium followed by injecting 3–5ml of 4% formalin into the left ventricular apex. Multiple organs (heart, lung, liver, kidney, spleen, brain) were harvested, fixed, cryo-sectioned, mounted, cross-stained with DAPI, and confocal imaging performed. To our surprise, the uptake in the heart was negligible, counter to our expectations based on the results of the alanine scan. Instead there was robust lung uptake of both peptides with little uptake into the liver or kidneys, and none in the heart (Figure 2: S7A top row; R11A middle row). In fact, lung uptake of R11A appeared to be even more robust than that of S7A. Decreasing doses of injected R11A still showed robust lung uptake even at the lowest dose of 1mg/Kg (Figure 2-bottom row; Supplemental Figure 1), with improving lung to liver ratios at lower doses of R11A (Supplemental Figure 1).

**Figure 3:**
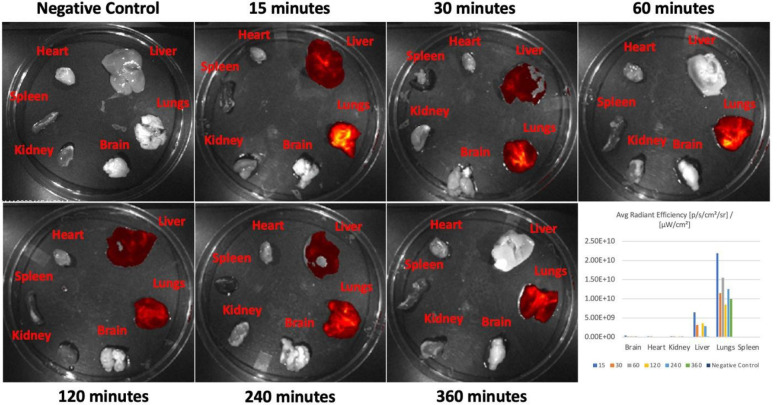
Ex-vivo imaging of multiple organs harvested from mice injected with Cy5.5 labeled R11A (5mg/Kg) and peptide allowed to circulate for indicated time points. There is immediate lung uptake peaking at 15 mins with peptide appearing in liver and decreasing over later time-points indicating predominantly biliary excretion of the peptide or it’s breakdown product(s).

In order to confirm uptake of these peptides by lung epithelial cells, a human bronchial epithelial cell line (CFBE41o-) was grown on coverslips, incubated with 10μM of S7A, R11A or a random, scrambled peptide (RAN) for 2, 10 and 30 minutes, washed with PBS, fixed, counterstained with DAPI and confocal microscopy performed. The two LTPs (R11A and S7A) were taken up at 10 minutes with uptake increasing at 30 minutes (Supplementary Figure 2). We next undertook a biodistribution study of R11A. Wild-type, 6-week old, CD1 mice were injected with 5mg/Kg of Cy5.5 labeled R11A peptide and allowed to circulate for 15, 30, 60, 120, 240, and 360 minutes. Mice were euthanized at the end of the circulation period, perfused and fixed as before, and multiple organs harvested and ex-vivo IVIS imaging performed immediately. Uptake by lungs was detectable and peaked at 15 minutes, the earliest time point tested. Fluorescence in liver decreased over time, with little fluorescence seen in kidneys, indicating primarily a hepatobiliary mode of excretion of the peptide or its breakdown products (Figure 3; Supplemental Figure 3, n=3).

**Figure 4:**
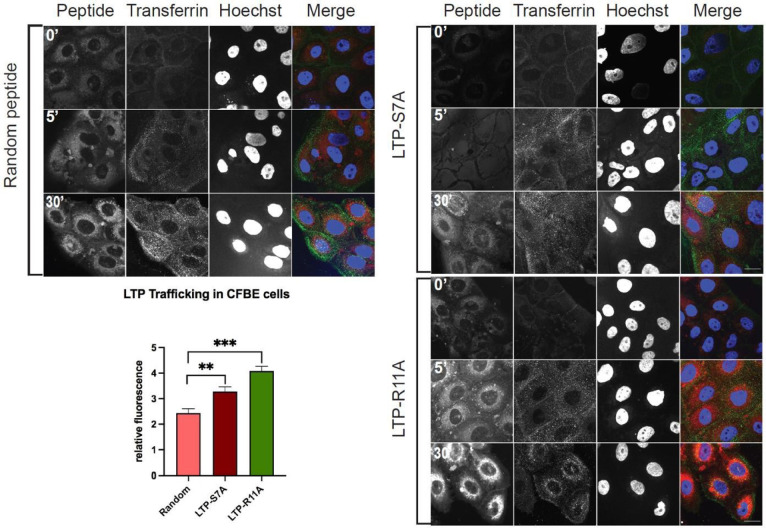
Bronchial epithelial cells take up LTPs in an energy independent, non-endocytic manner. Bronchial cells were serum-starved, followed by incubation with transferrin-488, or Cy5.5 labeled RAN, S7A, or R11A, followed by fixation at indicated time points, and confocal microscopy performed. Cells show very little uptake of the R11A and S7A peptide at 4°C, but increasing uptake at 5 and 30 minutes, without any co-localization with the green signal of transferrin that was endocytosed into cells. Red-Peptides; Green-Transferrin; Blue-Dapi. Scale bars represent 20μm.

In order to investigate if the mechanism of uptake involved endocytosis, human bronchial epithelial cells were grown on coverslips, serum starved for 1 hour at 37°C to stimulate endocytosis, washed with cold PBS before being transferred to 4°C and incubated with 488-labeled transferrin along with Cy5.5 labeled S7A, R11A or a RAN peptide for one hour. After this time period, cells were either fixed, counterstained with DAPI or transferred to 37°C for 5 or 30 minutes before being fixed, counterstained with DAPI and confocal microscopy performed. Our results show that the peptides were internalized and did not show co-localization with the green fluorescence of transferrin that had been endocytosed. These results indicate that the peptides are being taken up by the bronchial epithelial cells in a non-clathrin dependent endocytic process (Figure 4).

**Figure 5:**
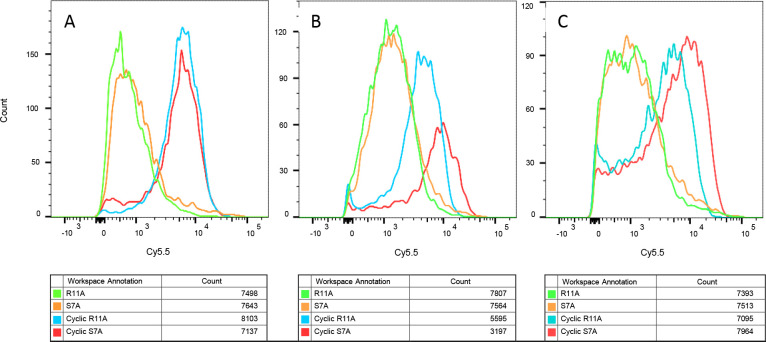
Cyclization of S7A and R11A increases transduction efficiencies by ~100-fold. Human bronchial cells were incubated with linear (1μM) or cyclic peptides (100nM) and yellow fluorescent live-dead stain, and intensity of Cy5.5 fluorescence evaluated on live cells (yellow-fluorescence negative). Cyclic peptides had a roughly 10-fold increased fluorescence as compared to the linear counterparts even though the latter were incubated at a 10-fold higher concentrations. Results shown are for three separate experiments with 10,000 cells/group evaluated for each experiment.

There have been multiple reports of cyclic versions of cell penetrating peptides having higher transduction abilities *in vivo* compared with their linear counterparts, likely due to better serum stability profiles^18–20^. In order to test whether this hypothesis applies to the present peptides, we synthesized cyclic versions of R11A and S7A by adding a lysine residue to the N-terminus, and performing a “head to tail” cyclization reaction of the terminal amino and carboxyl groups, prior to labeling the epsilon amine-group of lysine with Cy5.5. Human bronchial epithelial cell lines were incubated with linear (1μM) or cyclic peptides (100nM) for 30mins, along with a yellow live-dead stain, washed extensively with pre-warmed PBS, trypsinized and FACs performed. At 10-fold lower dose the cyclic peptides still showed a 10-fold higher cellular uptake, indicating a ~100-fold increase in transduction efficiencies (Figure 5).

**Figure 6:**
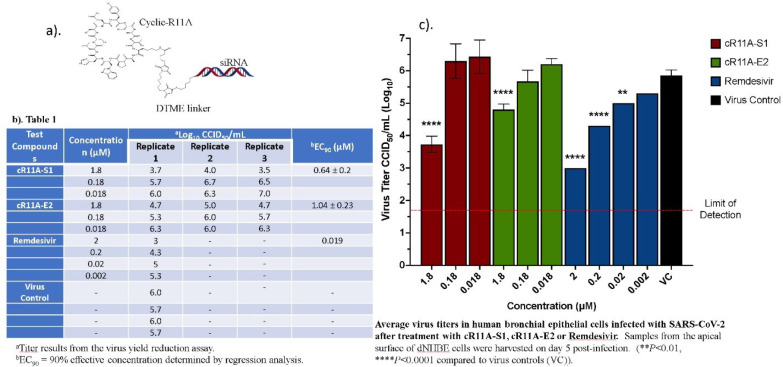
In vitro Antiviral Activity and EC90 Values for cR11A-siRNA-S1, cR11A-E2, and remdesivir against SARS-CoV-2 in human bronchial epithelial cell line. A). Structural representation of the cR11A along with the DTME linker linking to the siRNA represented by double-strands. B). Table showing viral titers from human bronchial epithelial cells treated with the cR11A-siRNA conjugates for 24 hours prior to infection with SARS-CoV-2 virus and the respective EC90 values, along with the EC90 value for remdesivir. C). SARS-CoV-2 viral titers plotted for cR11A-S1, cR11A-E2, and remdesivir against the virus only control. All infections and assays done in triplicate.

Next, as proof of concept, our aim was to utilize cyclic R11A (cR11A) to deliver siRNA targeting various structural proteins of the SARS-CoV-2 virus as possible anti-COVID therapy. In order to design SARS-CoV-2 specific siRNA targeting key viral structural proteins, spike S, envelope E and nuclear N proteins, we obtained SARS-CoV-2 complete genome sequence from GenBank (Accession Number: MN908947.3)^21^. We targeted gene-specific siRNA against SARS-CoV-2 structural proteins, and minimized off-target effects by utilizing multiple functional siRNA selection algorithms^22–25^ using siDirect2.0^26^. Two optimized siRNAs per target protein were selected based on thermodynamic stability^22^ (Supplemental Table 1). These siRNAs were synthesized with a terminal thiol group and then conjugated to the side amine group of cR11A via a siRNA mono-dithio-bis-maleimidoethane (DTME) intermediate (see methods for detailed protocol). DTME contains an internal disulfide bond and has been used to link multiple siRNAs together^27^. Our rationale was that cR11A would internalize the siRNAs, and the disulfide bond would subsequently be cleaved in the reducing intracellular environment thereby releasing the siRNA to knock down target viral mRNA of interest via the RISC complex, and arrest viral replication. The success of the conjugation was confirmed by MALDI-Tof analysis (Supplemental Figure 4). Internalization was confirmed in human bronchial epithelial cell lines before testing the top 5 (for transduction efficiencies) in VERO cell lines that were pre-treated with the cR11A-siRNA for 24 hours prior to infection with SARS-CoV-2 virus. Of the conjugates tested, cR11A-S1 and cR11A-E2, appeared to be most promising (Supplemental Table 2). Therefore, the antiviral activity of these two conjugates was evaluated further against SARS-CoV-2 (strain USA_WA1/2020) in a highly differentiated, three-dimensional, *in vitro* model of normal human bronchial cells (MatTek Corporation). These cells have unique properties in forming layers, the apical side of which is exposed only to air and has a mucin layer. Each cR11A-siRNA was tested for antiviral activity at 3 concentrations (1.8; 0.18; and 0.018 μM) in triplicate. cR11A-siRNA conjugates were applied to the apical and basal surface of cells for 24 hours prior to applying SARS-CoV-2 virus to the apical side only with incubation for 2 hours. Antiviral activity was measured by virus yield reduction assay in VERO 76 cells 5 days after infection. Remdesivir was included as a positive control and was tested at 4 concentrations (2, 0.2, 0.02, and 0.002 μM). As a virus only control, 3 of the cell wells were treated only with cell culture medium. Virus released into the apical compartment of the tissues was harvested and plated on VERO 76 cells for virus yield reduction titration. The virus yield results, EC_90_ values are summarized in Figure 6. Reduction of SARS-CoV-2 titers by cR11A-S1 and cR11A-E2 occurring at a concentration of 1.8 μM were statistically significant compared to virus controls. In human bronchial epithelial cells, reduction of SARS-CoV-2 titers by remdesivir at concentrations of 2, 0.2, and 0.02 μM were statistically significant compared to virus controls (Figure 6). cR11A-S1 inhibited virus replication with an EC_90_ value of 0.64 ± 0.2 μM, while cR11A-E2 inhibited virus replication with an EC_90_ value of 1.04 ± 0.2 μM, and remdesivir inhibited virus replication with an EC_90_ value of 0.019 μM (Figure 6b).

## Discussion

In this day and age of increasing air pollution, multi-drug resistant bacteria, and evolving new viral pathogens, novel vectors targeting lung epithelium with the ability to deliver myriad cargoes would be a major breakthrough. In this current body of work, we present two synthetic, non-naturally occurring lung targeting peptides, S7A and R11A. Although this was a serendipitous finding, in retrospect, changing the amino acid sequence in the terminal half of CTP would have changed its cardiomyocyte transduction ability as indicated by the study findings of CTP-A and CTP-B, which suggest that the transduction abilities of CTP lie in its C-terminus. To the best of our knowledge these two S7A and R11A peptides are first in their class of lung epithelial targeting peptides.

A nine amino acid long cyclic peptide, CARSKNKDC, termed CAR was reported as targeting multiple layers of pulmonary arteries in a rat model of pulmonary hypertension^28^, and was used to deliver micelles containing fasudil, an anti-pulmonary hypertension drug, to pulmonary endothelium^29^. This CPP uptake was limited to pulmonary endothelial cells, and did not target deeper lung tissue. In the pulmonary space, development of CPPs have been largely in the context of targeting lung cancers^30–32^. A strategy to target lung cancers takes advantage of the tumor microenvironment by targeting non-tissue specific CPPs to lung cancers by engineering CPPs that are activated in the tumor microenvironment. This was achieved by neutralizing the polycationic structure of these peptides by linking them to polyanionic peptides via a cleavable linker. These linkers were designed to take advantage of greater metalloproteinase 2 expression in the tumor microenvironment^33^, or greater oxidative stress leading to cleaving of the neutralizing peptide and unmasking of the CPP^34^.

Investigators have taken advantage of the accessibility of the lung by both systemic and inhalation routes, and have used non-cell specific penetrating peptides, like Tat peptide to deliver therapeutics of interest via the inhalational route^35^. Although the inhalational route has distinct advantages, like decreased dose requirement, less systemic side effects, and bypassing of the hepatobiliary system, it is not a feasible route in conditions like cystic fibrosis or primary ciliary dyskinesia. In these conditions’ patients are riddled by thick mucus secretions and significant defects in mucociliary clearance, leading to mucus accumulation and drug entrapment. Another factor to consider is that a large fraction of inhaled drug are deposited in the upper airways and may not reach the terminal bronchioles or alveoli. Additionally, the uptake of drugs may be limited to the very superficial bronchiolar epithelial layer. Our two LTPs show robust uptake diffusely by lung epithelial tissue after an intravenous injection, bypassing the drawback of entrapment in mucus layers.

In order to study the mechanism of transduction, and rule out endocytosis as the mechanism of uptake, we performed serum starvation studies on human bronchial epithelial cell lines. Serum starvation was followed by co-incubation of cells with fluorescently labeled transferrin (taken up by endocytosis) vs. S7A or R11A. There was no colocalization of transferrin with the labeled Cy5.5 of the peptides, ruling out clathrin-mediated endocytosis. This was encouraging as the inside of an endocytic vesicle is still outside of the cell, and peptides with their cargoes taken up by this mechanism are likely to be degraded, and not able to exert the desired therapeutic effect. We chose to follow detailed biodistribution of R11A in this body of work, in preference over S7A, as the former showed uptake even at the lowest 1mg/Kg dose with lung to liver ratios increasing with lowering of the dose. Lung uptake of R11A occurred in as little as 15 mins, which is similar to CTP, where peak uptake also occurred at 15 mins, the earliest time point tested^15^. We compared the cyclized version of both S7A and R11A to their linear counterparts in human bronchial epithelial cells lines *in vitro*, and found that cyclization increases uptake by almost 100-fold compared to the linear counterparts. Our findings are similar to that reported by others in the literature^36–39^, with increased transduction felt to be due to reduced conformational freedom, greater metabolic stability and higher resistance to proteolytic degradation. Due to the finding of R11A exhibiting better lung to liver ratios, and cyclic versions having significantly higher transduction efficiencies, cR11A was selected as the vector of choice to carry duplex siRNA, targeting various structural proteins of SARS-CoV-2 virus, conjugated to its N-terminus via a DTME linker. RNA interference as a therapeutic strategy against SARS-CoV-2 has been demonstrated by others^40–43^, utilizing lipid nanoparticles^44,45^, and peptide-dendrimers^46^. The advantage of our approach is use of a lung-specific CPP, its transduction capabilities further optimized via a cyclization strategy. Our proof-of-concept studies in human bronchial epithelial cell lines, grown in an air-liquid interface to mimic the bronchial environment, showed significant anti-viral activity for an siRNA targeting the spike protein and one targeting the envelope protein. Whether there is a synergistic effect with the use of both siRNAs, and at what doses, remains to be seen.

In this era of COVID pandemic, inhalational therapies that patients can self-administer if tested positive, or before the bronchitis/pneumonia becomes a systemic, life-threatening viremia, would be of immense value. However, in addition to testing the synergistic effects of the two identified siRNA, significant challenges will need to be overcome in the arena of aerosolized delivery of LTPs carrying siRNA. The aerodynamic diameter of the conjugates and their suspension medium would need to be 1–5μm^47^. There would be the potential for degradation of the peptide or the conjugate due to exposure to the shear force required for atomization. However, meeting these challenges could open up new forms of therapies for not just the current pandemic, but other viral pathogens like influenza A. Another advantage is the ability to swap the siRNAs for a sequence more conducive to emerging variants, since the chemistry for these 1:1 peptide to siRNA conjugation using a covalent, intracellularly degradable linker is now established, and hence can be applied to other CPPs and siRNAs.

In conclusion, this body of work has identified two novel lung targeting peptides that are able to deliver siRNA to human bronchial epithelial cell lines as a proof of their usefulness in delivering lung-specific therapeutics. We show that the lung is robustly transduced with lung to liver ratios improving with lower administered dose *in vivo*. We also show that cyclic versions of these peptides were more efficient than their linear forms. Our research has led to many questions that need to be addressed. In addition to aerosolization and potential inhalational route of delivery, the mechanism of transduction needs further study to identify potential binding partners for these unique peptides. This is not simply of academic interest as loading these peptides with cargoes could potentially mask the binding domain and interfere with cell penetration. Complete biodistribution and toxicity studies will need to be carried out for cR11A. Cyclic R11A was able to deliver siRNA duplexes, almost 10-fold greater than its own size, intracellularly and show a therapeutic effect *in vitro*. This ability to deliver cargoes many times their size in a functional form has been demonstrated before^6^. However, the cargo size limitation for LTPs, as there will be one, needs to be elucidated.

## Methods

### Peptide Synthesis:

**Linear Cy5.5 labeled lung targeting peptides (LTPs)** were synthesized on a Liberty CEM microwave synthesizer using Fluorenylmethyloxycarbonyl (FMOC) chemistry. Stepwise addition of each FMOC protected amino acid was accomplished on an amide resin as solid support using Ethyl-(*2Z*)-2-cyano-2-hydroxyiminoacetate/*N,N*-Diisopropylcarbodiimide (Oxyma/DIC) activation chemistry. The N-terminal amino group of the resin bound LTP peptides were then conjugated with Cy5.5-NHS (Lumiprobe Corporation) in DIPEA/DMF. Final cleavage of Cy5.5-LTP from the resin with Trifluoroacetic acid : Triisopropylsilane : H2O (TFA:TIPS:H2O-90:25:25) was followed by precipitation in Diethyl Ether (EtO2). The resulting crude Cy5.5-LTP peptides were purified by semi-preparative C-5 RP-HPLC on a Waters Delta Prep 4000 chromatography system using standard Acetonitrile/0.1%TFA gradient conditions.

MALDI-TOF analysis of the purified conjugates on an Applied Biosystems Voyager workstation using α-Cyano-4-hydroxycinnamic acid (CHCA) matrix allowed for confirmation of the expected mass and identity of the final product.

### Peptide Synthesis:

**Cyclic Cy5.5 labeled lung targeting peptides (LTPs)** were synthesized as above using 2-chlorotrityl resin as solid support. Cleavage of fully-protected peptide fragments were accomplished under mildly acidic conditions followed by head to tail cyclization using 3- (diethoxyphosphoryloxy)-1,2,3-benzotriazin-4(3H)-one (DEPBT) in Dimethylformamide/Dichloromethane (DMF/DCM) for 5 days at room temperature. Final cleavage and deprotection of the cyclized LTP peptides using Trifluoroacetic acid:Triisopropylsilane:H2O (TFA:TIPS:H2O - 90:25:25) was followed by precipitation in Diethyl Ether (EtO2) to isolate the crude peptide. The epsilon amino group of Lysine was then labelled with Cy5.5-NHS in 0.1M Triethylammonium bicarbonate (TEAbc)/acetonitrile at pH 8.5. The resulting Cy5.5 labelled cyclic LTP peptide was then directly purified by preparative C- 18 RP-HPLC on a Waters Delta Prep 4000 chromatography system using standard Acetonitrile/0.1%TFA gradient conditions followed by lyophilization. MALDI-TOF analysis of the purified conjugates on an Applied Biosystems Voyager workstation using α-Cyano-4-hydroxycinnamic acid (CHCA) matrix allowed for confirmation of the expected mass and identity of the final product.

### Peptide Synthesis:

**Cyclic thiolated lung targeting peptides (LTPs)** were synthesized as above using 2-chlorotrityl resin as solid support. Cleavage of fully-protected peptide fragments were accomplished under mildly acidic conditions followed by head to tail cyclization using 3- (diethoxyphosphoryloxy)-1,2,3-benzotriazin-4(3H)-one (DEPBT) in Dimethylformamide/Dichloromethane (DMF/DCM) for 5 days at room temperature. Final cleavage and deprotection of the cyclized LTP peptides using Trifluoroacetic acid: Triisopropylsilane:H2O (TFA:TIPS:H2O-90:25:25) was followed by precipitation in Diethyl Ether (EtO2) to isolate the crude peptide. The epsilon amino group of Lysine was then thiolated with 2-iminothiolane (Traut’s reagent) in 0.1M Triethylammonium bicarbonate (TEAbc)/acetonitrile at pH 8.5. The resulting thiolated cyclic LTP peptide was then directly purified by preparative C-18 RP-HPLC on a Waters Delta Prep 4000 chromatography system using standard Acetonitrile/0.1%TFA gradient conditions followed by lyophilization. MALDI- TOF analysis of the purified conjugates on an Applied Biosystems Voyager workstation using α- Cyano-4-hydroxycinnamic acid (CHCA) matrix allowed for confirmation of the expected mass and identity of the final product.

### Design of anti-SARS-CoV-2 siRNA:

To select a target gene-specific siRNA with minimized off- target effect against SARS-CoV-2 envelope E protein and nuclear N protein, siRNAs were designed by multiple functional siRNA selection algorithms using siDirect2.0. SARS-CoV-2 complete genome sequence and RNA sequence for E and N protein gene were obtained from GenBank (Accession Number: MN908947.3). We have selected the optimal 2 siRNAs per target, based on thermodynamic stability.

### Conjugation of siRNA to LTPs:

C6 protected siRNA oligomers (IDT technologies) were reduced to their free thiol form using DL-Dithiothreitol (DTT) in 0.1M Triethylammonium bicarbonate (TEAbc) at pH 8.5 and then reacted with dithio-bis-maleimidoethane (DTME) in 300mM NaOAc/acetonitrile at pH 5.2. Purification of the siRNA-DTME intermediate by precipitation with ethanol was followed by reaction with the purified cyclic R11A peptide in 300mM NaOAc/acetonitrile at pH 5.2 with gentle mixing at room temperature. Analytical C-18 RP-HPLC purification of the resulting siRNA-DTME-LTP-Cy5.5 conjugates using triethylamine acetate (TEAA)/Acetonitrile gradients on a Waters Alliance chromatography system was followed by lyophilization and then re-lyophilization from nuclease free water. MALDI-TOF analysis of the purified conjugates on an Applied Biosystems Voyager workstation using 3-hydroxypicolinic acid (3-HPA) matrix in ammonium citrate allowed for confirmation of the expected mass and identity of the final siRNA-cyclic-LTP conjugate.

### Fluorescence Activated Cell Sorting:

Human bronchial epithelial cell lines were passaged and plated into 6-well plates at a cell density of 100,000 cells/well. After being allowed to settle for at least 48 hours, media was aspirated and replaced with media containing 1μM or 100nM of linear or cyclic LTPs respectively. To each well, 1μL/ml of yellow Live-Dead stain was added as well. Cells were allowed to incubate for 30 mins, and then washed 3x with pre-warmed PBS, trypsinized, trypsin neutralized and cells collected by spinning down. After one wash with PBS, cells were fixed with 2% formalin at room temperature for 10 mins, formalin washed off and cells suspended in 500μL of PBS, placed into glass round bottomed tubes and FACS performed. Untreated cells, cells treated with Live-Dead stain alone, and cells treated with 1μM of a scrambled, random peptide were used as controls. All groups were tested in biological triplicates. Gating was performed after excluding dead cells in the Live-Dead channel.

### Anti-viral Testing of Cyclic R11A-siRNAs:

The antiviral activity of two cyclic small interfering RNAs (siRNAs) were evaluated against SARS-CoV-2 (strain USA_WA1/2020) in a highly differentiated, three-dimensional (3D), *in vitro* model of normal human bronchial (dNHBE) cells. Each siRNA was tested for antiviral activity at 3 concentrations (1.8; 0.18; and 0.018 μM) in triplicate 3D inserts of wells of dNHBE cells (MatTek Corporation). Antiviral activity was measured by virus yield reduction assay in Vero 76 cells 5 days after infection. Remdesivir was included as a positive control and was tested at 4 concentrations (2, 0.2, 0.02, and 0.002 μM). The cyclic siRNAs (cR11A-S1 and cR11A-E2) were previously received as a solid and dissolved in 100% DMSO at a concentration of 20 mg/mL. The compounds were further diluted to the test dilutions in the MatTek culture medium (AIR-100-MM). Differentiated normal human bronchial epithelial (dNHBE) cells were made to order by MatTek Corporation (Ashland, MA) and arrived in kits with either 12- or 24-well inserts each. dNHBE cells were grown on 6mm mesh disks in transwell inserts. During transportation the tissues were stabilized on a sheet of agarose, which was removed upon receipt. One insert was estimated to consist of approximately 1.2 × 10^6^ cells. Kits of cell inserts (EpiAirway^™^ AIR-100, AIR-112) originated from a single donor, # 9981, a 29-year-old, healthy, non-smoking, Caucasian female. The cells have unique properties in forming layers, the apical side of which is exposed only to air and that creates a mucin layer. Upon arrival, the cell transwell inserts were immediately transferred to individual wells of a 6-well plate according to manufacturer’s instructions, and 1 mL of MatTek’s proprietary culture medium (AIR-100-MM) was added to the basolateral side, whereas the apical side was exposed to a humidified 5% CO_2_ environment. Cells were cultured at 37°C for one day before the start of the experiment. After the 24 h equilibration period, the mucin layer, secreted from the apical side of the cells, was removed by washing with 400 μL pre- warmed 30 mM HEPES buffered saline solution 3 times. Culture medium was replenished following the wash steps. SARS-CoV-2 strain USA-WA1/2020 was passaged twice in Vero 76 cells to create the virus stock. Virus was diluted in AIR-100-MM medium before infection, yielding a multiplicity of infection (MOI) of approximately 0.0015 cell culture infectious dose, 50% endpoint (CCID_50_) per cell.

Treatment with cyclic R11A-siRNAs (120μL) was applied to the basolateral and apical side for 24 hours prior to infection. For the infection, virus (120) was applied to the apical side for a 2- hour incubation. Following the 2-hour infection, the apical medium was removed, and the basal side was replaced with fresh compound or medium. As a virus control, 3 of the cell wells were treated with cell culture medium only. As a cell control, 3 of the cell wells were treated with cell culture medium only. The cells were maintained at the air-liquid interface for 5 days after which the medium was removed and discarded from the basal side. Virus released into the apical compartment of the tissues was harvested by the addition of 400 μL of culture medium that was pre-warmed at 37°C. The contents were incubated for 30 min, mixed well, collected, thoroughly vortexed and plated on Vero 76 cells for virus yield reduction (VYR) titration. Triplicate and singlet wells were used for virus and cell controls, respectively.

Vero 76 cells were seeded in 96-well plates and grown overnight (37°C) to 90% confluence. Samples containing virus were diluted in 10-fold increments in MEM containing 2% fetal bovine serum and 50 μg/mL of gentamicin and 200 μL of each dilution transferred into respective wells of a 96-well microtiter plate. Four microwells were used for each dilution to determine 50% viral endpoints. After 5 days of incubation, each well was scored positive for virus if any cytopathic effect (CPE) was observed as compared with the uninfected control. The virus dose that was able to infect 50% of the cell cultures (CCID_50_ per 0.1 mL) was calculated by the Reed-Muench method (1938) and expressed as expressed as log_10_ CCID_50_/ml. Untreated, uninfected cells were used as the cell controls.

### Animal Studies:

All animal protocols were approved by the University of Pittsburgh Institutional Animal Care and Use Committee (IACUC). Wild-type, 6-week old, female CD1 mice were purchased from Charles Rivers. After at least 48 hours of acclimatization after arriving in the animal colony, mice were weighed and anesthetized with an intraperitoneal injection of a 1:1 cocktail of Ketamine:Xylazine at 1–2μL/gm body weight. After adequate anesthesia was achieved, mice were injected via tail vein with Cy5.5 labeled linear R11A and allowed to circulate for various time points. At the end of circulation time, mice were euthanized using inhalational CO2 as per IACUC-approved protocol, chest cavity opened, right atrium nicked and 3–5ml of 4% formalin injected through the left ventricular apex to perfusion fix the animals. Various organs were harvested, rinsed once in PBS and placed on a petri dish to be imaged using IVIS (Perkin- Elmer – S5 instrument). All mice were imaged using identical instrument settings (stage position, exposure time, binning, F-stop etc.). Various organs were placed in 4% Formalin at room temperature for 4 hours, transferred to 15% sucrose at 4°C overnight, then 30% sucrose at 4°C overnight, before being embedded in 50% OCT medium diluted with 50% 1x PBS, frozen using liquid nitrogen and stored, light-protected in −80°C for later cryosectioning. Organs were sectioned at 6 micron thickness, slides made, mounted with DAPI containing mounting medium and confocal microscopy performed. All time-points were tested in at least triplicate (n=3).

## Data Availability

All amino acid sequences of the peptides used are provided in the manuscript. All siRNA duplexes tested are provided in the supplemental material. Detailed methods on synthesis of linear and cyclic peptides, as well as conjugation of siRNA to lysine side N-terminus of cyclic peptides is given in sufficient detail to enable reader to replicate our results.

## Supplementary Material

Supplement 1
