# Supplementary material for: Novel Lung Targeting Cell Penetrating Peptides as Vectors for Delivery of Therapeutics
Source: Res Sq. 2021 Nov 15:rs.3.rs-1056707. Preprint. [Version 1] doi: 10.21203/rs.3.rs-1056707/v1 (PMC8609903; doi:10.21203/rs.3.rs-1056707/v1)
Supplement: Supplement 1 [file ff923b542dc6ceb96173749b.pdf]

Novel Lung Targeting Cell Penetrating Peptides as Vectors for Delivery of Therapeutics

Kayla McCandless, Sanjay Mishra, Jeffrey Stiltner, Kyle S. Feldman, Hisato Yagi, Ray Yurko, Kazi Islam, Jonathan Brown, Raymond Frizzell, Maliha Zahid

Supplemental Material

Supplemental Results

Supplemental Figure 1:

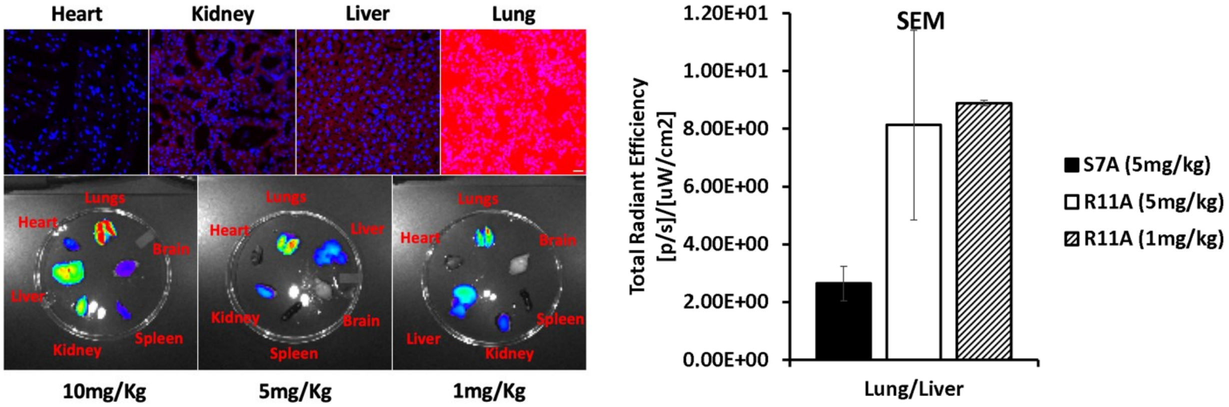

Supplemental Figure 1: Wild-type mice injected with 10mg/Kg of Cy5.5 labeled S7A or decreasing doses of R11A (10mg/Kg, 5mg/Kg and 1mg/Kg) R11A intravenously, euthanized at 15 mins, and multiple organs harvested for ex-vivo IVIS imaging followed by embedding, cryosectioning, counterstaining with DAPI and confocal microscopy. N=3 for each dose. Robust uptake of R11A by lung tissue is observed at even the lowest R11A dose of 1mg/Kg with lung to liver ratios improving consistently with lowering of the R11A dose.

Supplemental Figure 2:

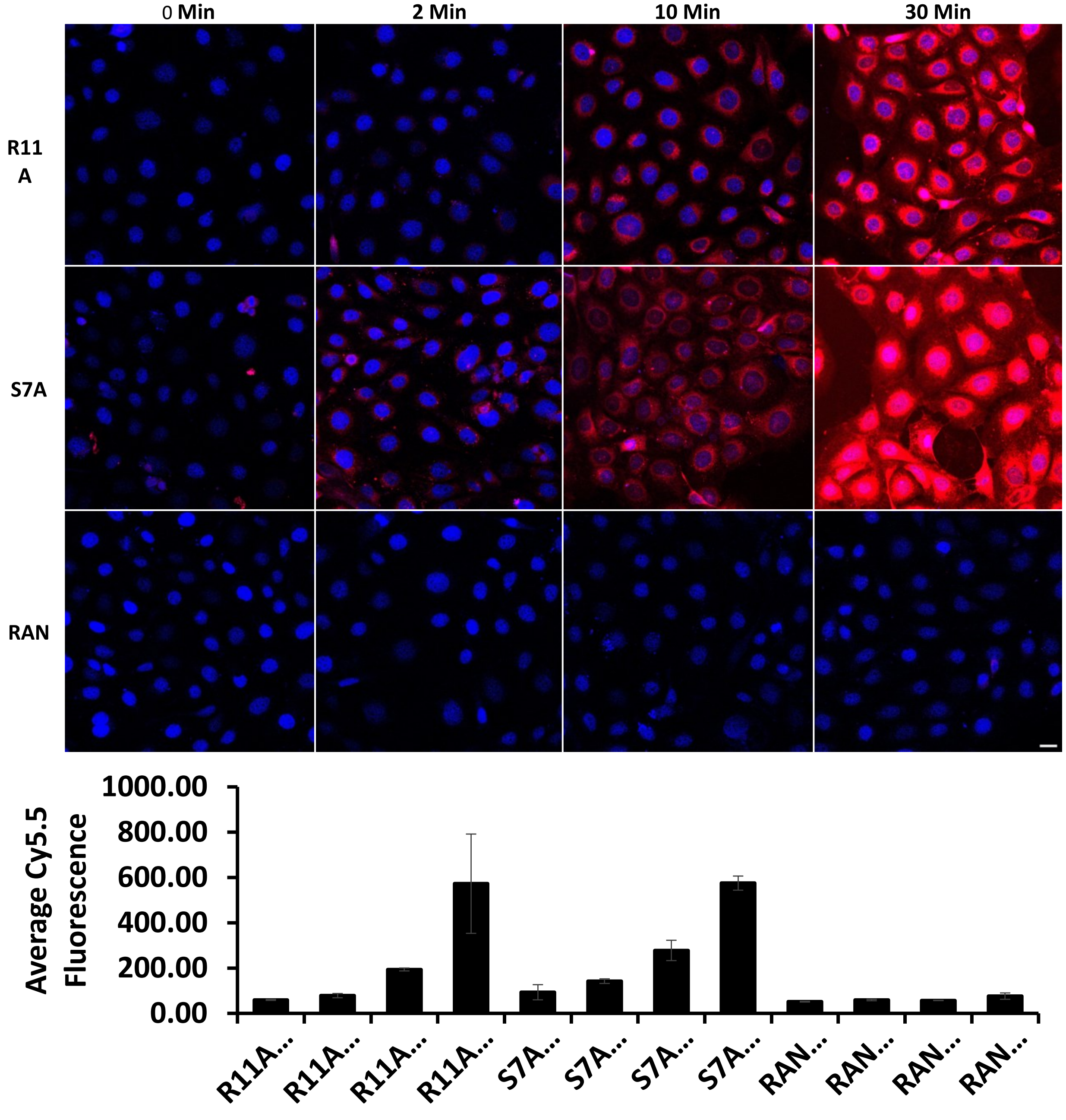

Supplemental Figure 2: Human bronchial epithelial cells transduced robustly with LTPs: Human bronchial epithelial cells were plated on cover slips and treated with 10µM of linear R11A, S7A or a scrambled random (RAN) peptide for indicated time points at 37°C, washed 3x with pre-warmed PBS, fixed, counterstained with DAPI and confocal microscopy performed. Both R11A and S7A are robustly internalized by cells by 30 mins, and appear to have a cytoplasmic, peri-nuclear localization. Random peptide has very little to no uptake.

Supplemental Figure 3:

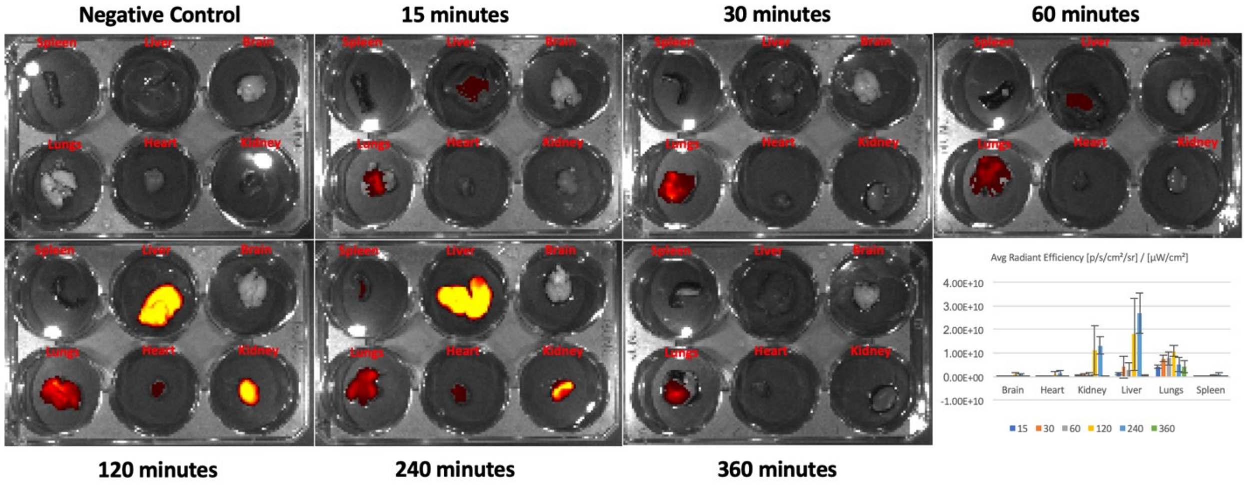

Supplemental Figure 3: Ex-vivo imaging of multiple organs harvested from mice injected with Cy5.5 labeled R11A (5mg/Kg) and peptide allowed to circulate for indicated time points. There is immediate lung uptake peaking at 60 mins with peptide appearing in liver at later time-points indicating predominantly biliary excretion of the peptide or it's breakdown product(s).

Supplemental Table 1: List of duplex siRNA and their targets tested in our study.

Target Protein	Target Position	Target Sequence	RNA oligo, Guide	Passenger	seed-duplex stabilty (Tm), guide	Passenger2	MW-Guide	MW-Passenger
Envelop-E1	70-92	gtggtattcttgctagttacact	UGUAACUAGCAAGAAUACCAC	GGUAUUCUUGCUAGUUACACU	14.3	14.5	6908	6833
Envelop-E2	149-171	gtcttgtaaaaccttctttttac	AAAAAGAAGGUUUUACAAGAC	CUUGUAAAACCUUCUUUUUAC	5.5	7.2	6996	6738
Nucleocapsid-N1	789-811	tgccactaaagcatacaatgtaa	ACAUUGUAUGCUUUAGUGGCA	CCACUAAAGCAUACAAUGUAA	13.5	11.8	6896	6892
Nucleocapsid-N2	1101-1123	gcctaaaaaggacaaaaagaaga	UUCUUUUUGUCCUUUUUAGGC	CUAAAAAGGACAAAAAGAAGA	5.5	-3.8	6725	7065
Spike-S1	977-999	ttgttagatttcctaatattaca	UAAUAUUAGGAAAUCUAACAA	GUUAGAUUUCCUAAUAUUACA	-8	6.9	6918	6825
Spike-S2	2260-2282	ttgcaatatggcagtttttgtac	ACAAAAACUGCCAUAUUGCAA	GCAAUAUGGCAGUUUUUGUAC	5.6	5.6	6892	6896

Supplemental Figure 4: MALDI-Tof analysis of cyclic R11A-siRNA-S1 showing the size and peaks of the conjugate.

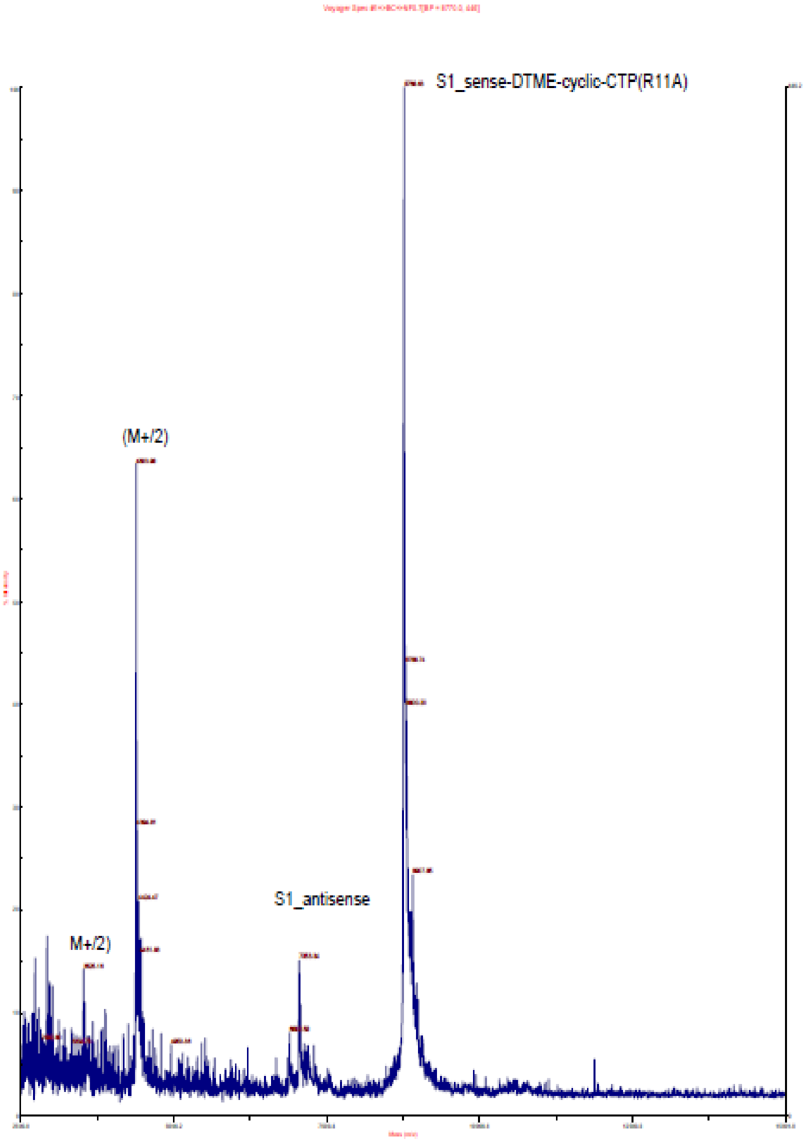

Supplemental Table 2: Results of VERO Cells incubated with cyclic R11A-siRNA conjugates followed by infection with SARS-CoV-2 virus.

**Table 2a. Percent toxicity of University of Pittsburgh compounds on Vero 76 cells**					
	**Percent Toxicity**				
Conc. (μM)	**cR11A-S1**	**cR11A-S2**	**cR11A-E2**	**cR11A-N1**	**cR11A-N2**
100	0.0%	14.4%	9.7%	0.0%	4.5%
10	10.1%	2.8%	17.9%	0.0%	3.0%
1	18.1%	2.8%	17.9%	0.0%	5.2%
0.1	14.6%	2.8%	17.9%	0.0%	0.0%

**Table 2b. Percent cytopathic effect of University of Pittsburgh compounds against SARS-CoV-2**					
	**Percent CPE**				
Conc. (μM)	**cR11A-S1**	**cR11A-S2**	**cR11A-E2**	**cR11A-N1**	**cR11A-N2**
100	57.9%	64.6%	53.7%	78.6%	60.2%
10	87.3%	89.3%	89.6%	96.9%	90.8%
1	90.1%	93.1%	95.4%	97.6%	96.8%
0.1	90.1%	100.0%	100.0%	95.5%	99.3%
CPE - Cytopathic effect					
Compounds pretreated on cells for 24 hours prior to infection with SARS-CoV-2 virus.					

Supplemental References

Reed, L.J., Muench, H., 1938. A simple method of estimating fifty percent endpoints. The American Journal of Hygiene 27, 493–497.
